# The effect of self-reported knee instability on plantar pressure and postural sways in women with knee osteoarthritis

**DOI:** 10.1186/s13018-021-02823-6

**Published:** 2021-11-17

**Authors:** Liana Chaharmahali, Farzaneh Gandomi, Ali Yalfani, Alireza Fazaeli

**Affiliations:** 1 Sports Injuries and Corrective Exercises Department, Faculty of Physical Education and Sport Sciences, Razi University, Kermanshah, Iran; 2 Sports Injuries and Corrective Exercises Department, Faculty of Physical Education and Sport Sciences, Bu Ali Sina University, Hamedan, Iran; 3 Rheumatology Department, Shahid Beheshti Hospital, Hamedan University of Medical Sciences, Hamedan, Iran

**Keywords:** Knee osteoarthritis, Knee instability, Biodex, Postural control, Fall

## Abstract

**Background:**

Giving way and knee instability are common problems in patients with knee osteoarthritis, disrupting the daily activities and balance of the affected individual. The present study aimed to evaluate the postural control status of women with knee osteoarthritis with and without self-report knee instability (KI).

**Methods:**

This cross-sectional, single-blind study was conducted on 57 female patients with knee osteoarthritis. The patients were selected based on the inclusion and exclusion criteria and divided into two groups of with KI (*n* = 26) and without KI (*n* = 31). Fear of movement was assessed using the Tampa questionnaire, the degree of knee instability was measured based on the Fitzgard scale, the static and dynamic balance of the subjects were evaluated with open and closed eyes using a Biodex balance device, and foot pressure distribution situation was measured using a FDM-S-Zebris device.

**Results:**

Mean comparison showed a significant difference between the subjects with and without KI in static balance only in anterior–posterior direction with open eyes (*p* = 0.01) and closed eyes (*p* = 0.0001). In the dynamic balance test, the subjects in both groups had significant differences in terms of all the indicators of anterior–posterior stability (*p* = 0.001), medial–lateral stability (*p* = 0.0001), and overall stability (*p* = 0.0001) with closed eyes. However, no significant difference was observed with open eyes (*p* > 0.05). Multiple regression also indicated significant positive correlations between pain intensity and disease duration with the degree of KI (*p* < 0.05).

**Conclusions:**

According to the results, there were significant differences between the mean pain scores, static and dynamic balance, and the rate of fall between the women with knee osteoarthritis with and without the KI index. Therefore, patients with knee osteoarthritis, which also has an index of KI, are more susceptible to falls, and proper strategies are required to reduce the level of KI in these patients.

## Introduction

Giving way and knee instability (KI) during weight-bearing activities are common problems in patients with knee osteoarthritis (KOA). According to the literature, 60–80% of patients with KOA complain of recurrent KI [1, 2]. Knee instability is defined as a feeling of giving way, shifting, buckling or a sudden decrease in postural control while bearing weight [3, 4]. Feeling unstable could reduce the capacity to perform daily activities and is associated with poor physical function and pain [5]. KOA is associated with a 50–60% reduction in the quadriceps torque possibly due to atrophy and arthrogenic muscles inhibition [6]. The comparison of patients with knee pain with subjects without knee pain for 30 months has shown significant quadriceps muscle weakness [7].

Postural control as a key factor in preventing falls in these patients is defined as the ability to maintain the center of mass within the base of support in an upright position [8]. Optimal postural control helps individuals perform daily activities at an appropriate and safe level and prevents severe injuries due to falls, especially in patients with KOA [9]. Postural control is affected by the proper functioning of various body systems in the form of coordinated neuromuscular responses to determine the body position of the individual in the static and dynamic states [9]. The stability of the knee joint during movement is affected by the function of inactive structures (e.g., ligaments & joint capsules) and motor control (e.g., muscle contractions, neuromuscular control, and the sensory system) [10, 11].

Neuromuscular disorders such as muscle weakness [12, 13], impaired proprioception [14, 15], joint laxity [5], and knee instability [11] in patients with KOA may decrease postural control [16]. In addition, poor neuromuscular control to maintain balance in these patients alters their gait pattern and may accelerate disease progression by increasing the load on the knee [16–18]. Previous findings have confirmed that the improvement of knee stability could increase balance in patients with KOA and reduce the disease symptoms [6, 19].

According to a study in this regard, neuromuscular changes resulting from aging lead to the dysfunction of the sensory receptors and muscle weakness, which in turn impair postural control [20]. A systematic review also indicated that patients with KOA were more likely to have postural instability compared to healthy individuals. The difference is most often observed with closed eyes and may result from pain, impaired joint sense, and muscle weakness (especially quadriceps). Knee pain could inhibit the activity of the muscles around the joints, thereby affecting motor responses while controlling a position [6, 17, 18].

Several studies have assessed the difference in postural control between patients with KOA and healthy individuals, acknowledging the lower balance of KOA patients compared to healthy subjects. In this regard, Schrijvers et al. investigated the pattern of muscle activation and stability of the knee joint in patients with self-reported KI, patients reporting stable knees, and healthy individuals. In the KI group, the angle of knee flexion was high during the stance peak, late stance, early swing, and mid-swing phases in response to various external disturbances [10]. In another study, Sanchez-Ramirez et al. evaluated the correlations between postural control, muscle strength, proprioception, self-reported KI, and performance of patients with KOA, reporting decreased postural control, muscle weakness, and proprioception impairment in those with self-reported instability [16].

Due to the significant association of KOA and balance disorders, the effects of balance and postural control on the daily functions of these patients should be thoroughly studied, along with the frequent occurrence of self-reported KI. Fractures increase in KOA patients, and such instabilities instill the fear of movement in these individuals, thereby indirectly disrupting their daily functioning. So, the present study aimed to evaluate the balance status of KOA patients with and without self-report KI. The research hypotheses were: (1) There is a significant difference in the static and dynamic balance with open and closed eyes between the KOA patients with and without self-report KI; (2) there are significant differences in the time of foot contact with the ground, the length of gait line, pain intensity, fear of movement, and risk of falling between the KOA patients with and without self-report KI; (3) there are correlations between pain intensity and disease duration with the rate of self-report KI in the women with KOA.

## Materials and methods

### Participants

**Fig. 1:**
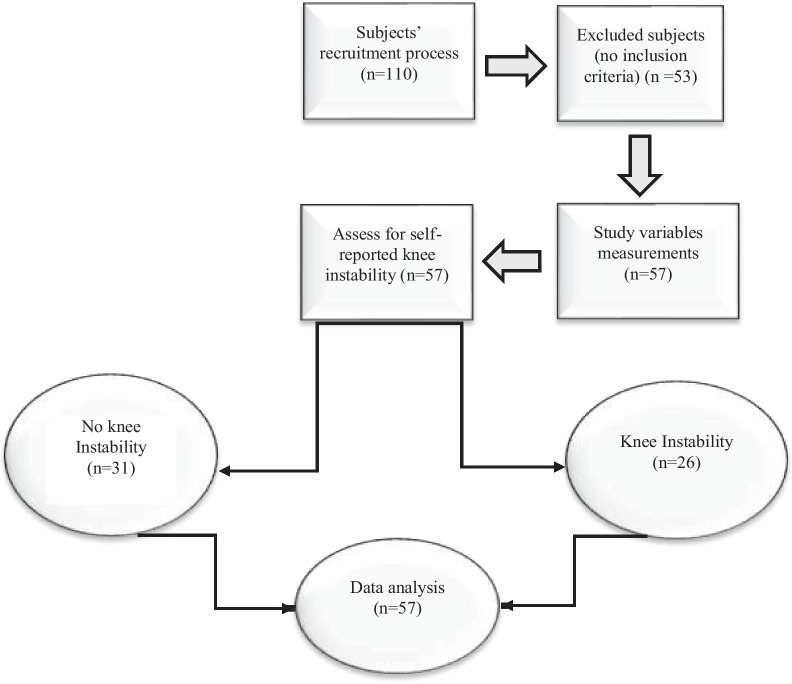
Study flow chart

This assessor-blind, cross-sectional study was conducted during February 3–March 15, 2021 at Bu-Ali Sina University in Hamadan, Iran. In a subspecialty clinic in Hamadan, 110 patients with KOA were identified with the definitive diagnosis of a rheumatologist, and 57 patients were enrolled in the study based on the inclusion and exclusion criteria. The inclusion criteria were age of more than 40 years (American College of Rheumatology clinical criteria for KOA, 1986), a minimum score of two on the Kellgren and Lawrence radiographic disease severity scale, and no regular exercise during the week. Patients with a history of arthroplasty, knee genu varum (distance between the internal epicondyles of the femur: ≥ 2.5 cm), genu recurvatum, stroke, uncontrolled muscular hypertension, obesity (BMI > 40 kg/m^2^), neuromuscular diseases (e.g., Parkinson's disease, MS), lower-extremity joint fractures, back/thigh pain, and concomitant femoral osteoarthritis were excluded from the study. In all the patients, the knee with the most difficulty was used for further assessments (Fig. 1).

### Procedures

The researcher was blinded to the procedures after the initial examination of the subjects in the rheumatology clinic, collecting the basic data (age, disease status, pain intensity, rate of fall, disease duration), and evaluating the inclusion and exclusion criteria. After obtaining written informed consent from the patients, they were referred to the Sports Rehabilitation Laboratory of Bu-Ali Sina University for further studies. Pain intensity, self-reported KI, knee flexion range of motion, static and dynamic balance, kinesiophobia, and plantar pressure distribution were assessed in all the participants.

#### Pain intensity and knee flexion range of motion (ROM)

The VAS is used to measure the intensity of perceived pain. This ruler is a horizontal strip with the length of 10 cm, one end of which is zero to show no pain, and the other end is 10 to show the most severe pain. The ruler also has two qualitative and quantitative sides. In the present study, the patients were asked to mark the qualitative side of the ruler based on their perceived pain intensity. Following that, the researcher turned over the ruler and recorded the marked point which was considered to be the severity of the patient's pain. The VAS is the most reliable pain rating system for the comparison of different periods and has been widely used in research studies. Its validity and reliability are excellent, and its internal correlation coefficient reliability has been reported to be ICC = 0.91 [19].

Passive knee flexion ROM was measured in degrees using a goniometer with the patient in the prone position on the test table during the physical examination. To measure knee flexion ROM, the participant was instructed to flex the knee without pain as far as possible, and the ROM was determined by the examiner, who also monitored for compensatory movement through the lower extremity and pelvis (21).

#### Static and dynamic balance

The Biodex balance system (BBS; Biodex Medical System Inc., Shirley, NY) assesses a person’s balance. In simpler terms, it measures the static standing balance and dynamic standing balance of an individual. The machine consists of a circular platform and a display unit. The subject stands on the circular platform, which tilts up to 20° in each direction (range of motion: 360°), and the platform tilts based on the level set through the display unit. The device has 12 levels of platform tilt, with level 12 offering the most stable platform (maximum resistance) and level one representing the most unstable platform (minimum resistance). In the present study, the following set of platform settings was used in a random order for all conditions.

**Fig. 2:**
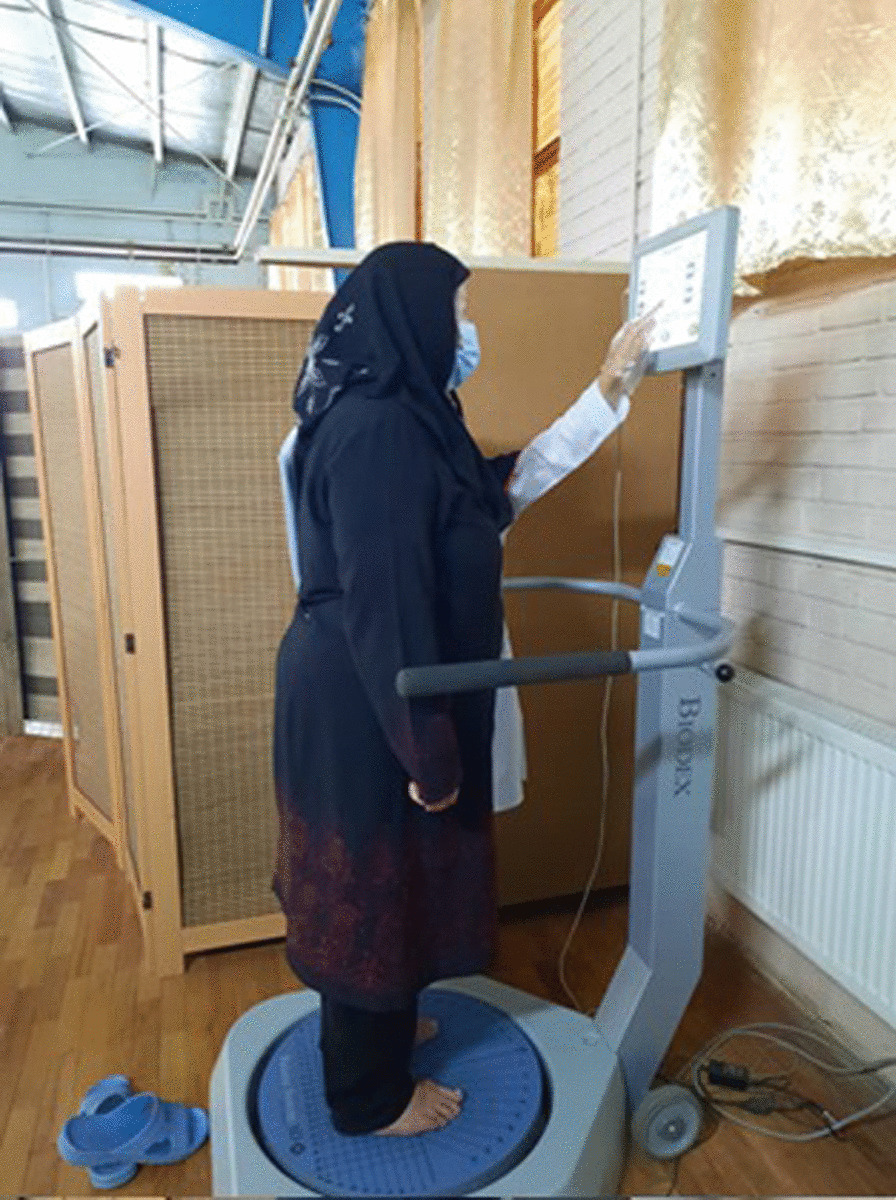
Evaluation of static and dynamic balance using biodex balance system

1. *Static balance* (fall risk: static): no platform movement
2. *Dynamic balance* (fall risk: 12 to 8): each test trial starts from dynamic level 12 and decreases to level eight (Fig. 2) [20].

The balance plate moves anteriorly posteriorly (AP) and medially-laterally (ML) and has three types of outputs, including the AP stability index (APSI), ML stability index (MLSI), and overall stability index (OSI).  These indices represent the standard deviations indicating the fluctuations around the reference point (a firm, horizontal platform) and are calculated by measuring the amount of time for which the platform has been deviated, along with the angulation degree of the deviation from the reference point. MLSI represents the center of the pressure displacements occurring on the *X* axis (medial–lateral axis) for both feet simultaneously. APSI represents the center of the pressure displacements occurring on the *Y* axis (anterior–posterior axis) for both feet simultaneously. OSI is a composite of the APSI and MLSI and represents the body sway on both the *X* and *Y* axes. OSI, MLSI, and APSI are calculated using the following equations:

$$OSI=\frac{\left(0-Y\right)2+(0-X)2}{\mathrm{Number\,of\,samples}}$$

$$\mathrm{MLSI}=\frac{(0-X)2}{\mathrm{Number\,of\,samples}}$$

$$\mathrm{APSI}=\frac{\left(0-Y\right)2}{\mathrm{Number\,of\,samples}}$$

In the center of balance (i.e., the position where the participants stood balanced as shown by a dot in the middle of the screen) are the variables *Y* and *X* (0.0). As the user deviates from the center of balance on the sagittal plane, the *X* value increases, while when they deviate from the center of balance on the frontal plane, the *Y* value increases. In other words, *X* and *Y* represent the coordinates of the center of gravity on the platform, and their value ​​at time (0.0) is t = 0. In the present study, we used the three APSI variables, MLSI, and OSI.

The patients were asked to stand barefoot on the BBS in front of the monitor, once with their eyes open and once with their eyes closed (both static and dynamic tests) and their hands dangling to their sides. If support groups were used during the experiment, those experiments would be discarded. The distance between the heels was kept constant to avoid the effects of compatibility on the stabilizing response due to different heel distances. At the next stage, the patients were asked to stand straight without holding their foot position and hold the moving black dot in the center of the cross on the monitor. For each condition, two experiments were performed (duration: 30 s each) with a 10-s rest interval [22].

#### Tampa scale of kinesiophobia (TSK)

To measure the fear of movement (kinesiophobia), we used the Tampa scale, which consists of 17 items. The total score is within the range of 17–68, which is calculated after reversing items four, eight, 12, and 16. Higher scores in the Tampa scale indicate a greater fear of movement due to pain perception. In this calculation, the score of 37 is distinguished by high and low scores. Notably, the Persian version of this questionnaire was developed and validated in 2010 by Jafari et al. (ICC = 0.68) [23].

#### Plantar pressure distribution

Data on foot pressure were obtained using the FDM-S plantar pressure device (ZEBRIS GmbH, Isny, Germany; ICC = 0.91) [24]. The device has dimensions of 54 by 34 cm, 2,560 high-sensitivity sensors, and a sampling rate of 50 Hz. The pressure plate was embedded in a wooden path of the same color as the pressure plate, and with the normal gait of the patients, the test was stopped once by the right foot and once by the left foot. In order to keep the patients' heads up and prevent extra movements, the subjects were asked to look at a specified vision target placed two meters apart during the test.

Three tests were undertaken from each patient (duration: 20 s each), and the subjects had a two-minute break between the repetitions. The mean results of the three tests were recorded as the test results of each patient [25]. In addition, the parameters of the length of the gait line (mm) and contact time (seconds) were also derived, and all the subjects walked over the FDM-S pressure mat[26].

#### Self-reported knee instability

To prevent the effect of bias on the assessments, the severity of knee instability was measured at the end of the other assessments. As mentioned earlier, the patients were divided into two groups of with knee instability (*n* = 26) and without knee instability (*n* = 31) based on their response to a six-point numerical scale ("*How much does emptying, slipping, or shifting your knee affect your daily activity?*"). Table 1 shows the definitions of the six levels of instability. The unstable group of patients included those manifesting signs of instability affecting their ability to perform daily activities (score 3), while the stable group included those reporting no instability or not perceiving the associated signs that affected their daily activities (scores > 4; ICC = 0.72) [27].

**Table 1:** Severity of complaints of self-reported KI taken from the Knee Outcome Survey-Activities of Daily Living Scale [26]

Scale	Self-reported KI
0	The symptom prevents me from all daily activity
1	The symptom affects my activity severely
2	The symptom affects my activity moderately
3	The symptom affects my activity slightly
4	I have the symptom but it does not affect my activity
5	I do not have giving way, buckling, or shifting of the knee

#### Ethical considerations

Written informed consent was obtained from the participants, and the study protocol was approved by the Ethics Committee of Razi University of Kermanshah (ethics code: IR.RAZI.REC.1400.006).

### Statistical analyses

Mean and standard deviation were used to report the descriptive statistics of the demographic variables (age, height, weight, BMI), and Shapiro–Wilk test was used to assess the normality of the data. In addition, Levene's test was applied to evaluate the homogeneity of the data, and independent samples t-test was used to compare the mean values between the study groups (KOA with KI and KOA without KI). Data analysis was performed in SPSS version 22 (Chicago, USA) at the significance level of *p* < 0.05 and 95% confidence interval (CI). Multiple regression was also employed to determine the effects of variables on each other. Effect size (ES) was calculated using the following formula:

$$ES=\frac{{t}^{2}}{{t}^{2}+(n1+n2-2)}$$

ES with *d* < 0.2 was considered to be small, while *d* > 0.5 was moderate, and *d* > 0.8 was considered large [28].

## Results

In total, 57 women with KOA were enrolled in the study (26 KOA with KI and 31 KOA without KI). Table 2 shows the descriptive statistics of the subjects. According to the results of Shapiro–Wilk test, the assumptions of the normal distribution and homogeneity of the variances were established (*p* > 0.05). According to the information in Table 2, the subjects in the study groups had no significant differences in terms of demographic characteristics (*p* > 0.05).

**Table 2:** Demographic characteristics of participants and the study parameters at baseline

Variables	Without self-reported KI (*n* = 31)	With self-reported KI (*n* = 26)	Significance (*p* value)
Age (years)	54.43 ± 6.45	53.70 ± 6.94	0.66
Female, n (%)	63 (100%)	0 (0%)	-
Height (cm)	158.67 ± 6.98	158.63 ± 5.55	0.98
Weight (kg)	78.99 ± 11.58	76.98 ± 9.19	0.45
Body mass index (kg/m^2^)	32.03 ± 3.76	30.62 ± 2.99	0.09
Self-reported knee stability scale (0–5)	2.18 ± 1.20	5.00 ± 0.00	0.0001*
Pain score (0–10)	8.40 ± 0.71	7.41 ± 0.51	0.0001*
Falling (n)	0	1.22 ± 1.08	0.0001*
TSK (17–68)	44.25 ± 3.71	44.35 ± 2.28	0.89
Knee flexion ROM (degree)			
Right leg	83.06 ± 9.36	77.70 ± 9.98	0.036*
Left leg	86.36 ± 8.92	78.23 ± 11.58	0.003*

^*^Values are mean ± standard deviations unless indicated otherwise. *p < 0.05

^†^All patients had to have at least a grade 2 knee OA to be included in the study

KI: knee instability; TSK: Tampa scale of kinesiophobia; ROM: range of motion

### Pain, kinesiophobia, and self-reported KI

Two independent sample t-tests indicated significant differences in the pain intensity (t_61_ = 6.34; 95% CI: 0.67, 1.29; *p* = 0.0001), degree of knee self-report instability (t_61_ = -13.01; 95% CI: -3.24, -3.38; *p* = 0.0001), and number of falls (t_61_ = 6.28; 95% CI: 0.83, 1.61; *p* = 0.0001) between the study groups (KOA with and without self-reported KI). Meanwhile, no significant difference was denoted in the fear of movement between the two study groups (Table 2).

### Static and dynamic balance

**Fig. 3:**
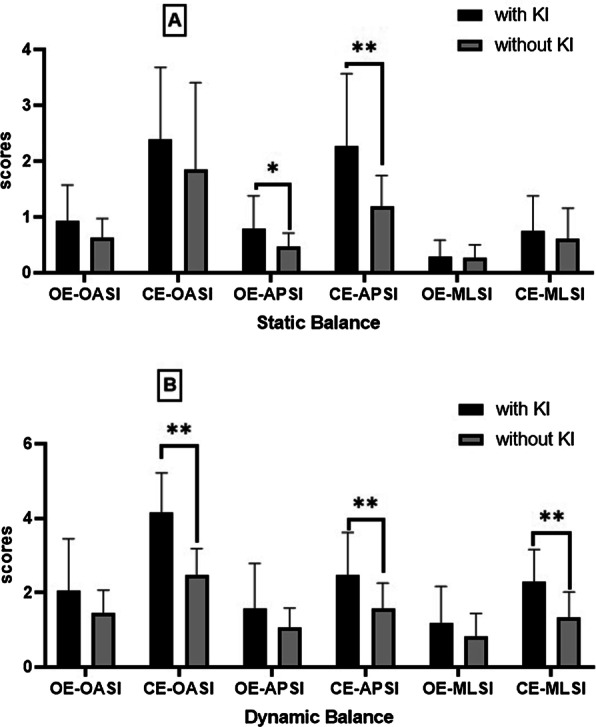
Comparison of mean scores of **a** static and **b** dynamic balance indices between patients with KOA with and without knee instability (*significant at 0.05, **significant at 0.01)

The balance state of the subjects was evaluated in the static and dynamic states with open and closed eyes, and the comparison of means indicated that the subjects with the characteristic of self-reported knee instability in the static balance state were significantly different only in terms of the anteroposterior stability with open eyes (t_61_ = 2.61; 95% CI: 0.07, 0.57; *p* = 0.01). Furthermore, a significant difference was denoted in this regard with closed eyes (t_61_ = 4.00; 95% CI: 0.54, 1.64; *p* = 0.0001). In the dynamic balance test of the subjects, significant differences were observed in both groups in terms of all the indicators of anteroposterior stability (t_61_ = 3.52; 95% CI: 0.38, 1.41; *p* = 0.001), medial–lateral stability (t_61_ = 4.63; 95% CI:0.14, 3.54; *p* = 0.0001), and overall stability (t_61_ = 6.87; 95% CI 1.19, 2.17; *p* = 0.0001), while no significant difference was denoted with open eyes (*p* > 0.05) (Table 3; Fig. 3).

**Table 3:** Summary of group differences in static and dynamic balance scales and foot pressure distribution

Variables	With instability (*n* = 26)	Without instability (*n* = 31)	*p* value	η2
	M (SD)	M (SD)		
Static				
Open eye				
APSI	0.78 (0.59)	0.46 (0.24)	0.01*	0.11
MLSI	0.28 (0.30)	0.26 (0.23)	0.8	0.001
OSI	0.92 (0.64)	0.75 (0.74)	0.37	0.01
Close eye				
APSI	2.27 (1.29)	1.18 (0.56)	0.0001*	0.22
MLSI	0.75 (0.61)	0.60 (0.55)	0.32	0.01
OSI	2.39 (1.28)	1.84 (1.56)	0.14	0.03
Dynamic				
Open eye				
APSI	1.56 (1.21)	1.07 (0.51)	0.06	0.06
MLSI	1.19 (0.97)	0.83 (0.60)	0.1	0.04
OSI	2.66 (0.38)	1.45 (0.61)	0.14	0.03
Close eye				
APSI	2.47 (1.14)	1.57 (0.51)	0.001*	0.18
MLSI	2.30(0.86)	1.33 (0.67)	0.0001*	0.28
OSI	4.15 (1.06)	2.48 (0.71)	0.0001*	0.46
Foot pressure distribution (dynamic)				
Gait line (mm)				
Right	218.53 (32.86)	225.90 (68.53)	0.6	0.001
Left	253.31 (114.44)	212.23 (60.28)	0.11	0.04
Contact time (s)				
Right	1.19 (0.45)	1.03 (0.26)	0.13	0.03
Left	1.36 (0.66)	1.28 (0.63)	0.62	0.001

All analyses were presented by mean (standard deviation)

M: mean; SD: standard deviation; APSI: anterior–posterior stability index; MLSI: medial–lateral stability index; OSI: overall stability index; η^2^: effect size

### Foot pressure distribution

In addition to assessing the static and dynamic balance of the KOA patients with and without knee instability, some indicators of the distribution of their foot pressure while walking were also evaluated. According to the obtained results, the two groups had no significant differences in the scale of right and left foot line length and duration of foot contact with the ground (*p* > 0.05) (Table 3). On the other hand, the multivariate regression indicated significant correlations between pain intensity, disease duration, and the degree of knee instability in the patients with KOA (*p* < 0.05). Correspondingly, increased pain intensity and disease duration were associated with increased knee emptying (Table 4).

**Table 4:** Results of multiple regression analyses detecting significant interaction relation-ships between pain and disease duration with knee instability scores

Variables	Beta (95% CI)	Standard beta	Significance (*p* value)
Age (years)	0.007 (− 0.04, 0.05)	0.03	0.76
VAS (0–10)	− 0.65 (− 1.12, − 0.18)	− 0.29	0.008 *
Duration (years)	− 0.21 (− 0.30, − 0.11)	− 0.49	0.0001*

CI: confidence interval; VAS: visual analog scale

*p < 0.05

## Discussion

Most patients with KOA complain of a feeling of giving way, buckling, and shifting in the knee to the sides, which is accompanied by a sudden decrease in the patient's postural control due to the locking and instability of the knee while bearing weight. This could be associated with a lack of trust in the knee joint and increase the risk of falls in these patients. The current research aimed to investigate the correlation between the KI rate in the women with KOA in the static and dynamic balance states and measure gait indices.

According to the obtained results, the static balance of the women with KOA and KI at the APSI level with open and closed eyes was significantly lower compared to the women with KOA without KI. In the dynamic balance state, the patients with KI were significantly different from the group without KI in terms of the balance indices APSI, MLSI, and OASI only with closed eyes. Therefore, the first hypothesis of the research was confirmed.

According to the results of the present study, the patients with and without self-report KI differed regarding the indicators of the dynamic balance state with closed eyes, which is in line with the previous findings in this regard, which have demonstrated that vision plays a pivotal role in maintaining balance [29]. In addition to the present study, this phenomenon has been confirmed in patients with osteoarthritis in a previous research conducted by Truszczyńska-Baszak et al. who limited the vision of KOA patients and observed that their postural control severely reduced, and training was required to maintain their balance control [30].

Another important source of situational information is the proprioception of the joints, which is impaired in these patients due to pain and the inhibition of the mechanical receptors (mechanoreceptors). As a result, patients' balance will be challenged more. According to our findings, closing the eyes and disconnecting the main sensory source to maintain balance caused most of the received information to be provided by the sense of depth, and the patients with KI had an impaired proprioception and a weaker kinesthesia compared to those without KI [6, 31]. In line with this finding, Kim et al. (2011) reported that patients with moderate-to-severe KOA relied more on their sense of sight to compensate for their postural instability. In addition, factors such as decreased quadriceps strength, impaired proprioception, and increased pain were reported to increase postural instability and decrease balance in these patients [32]. On the other hand, Wegner et al. (1997) stated that patients with knee osteoarthritis had increased postural sways in the anterior–posterior direction [33]. Furthermore, Hunt et al. (2010) observed a high level of hamstring and quadriceps muscle contraction at a more advanced level of the disease, which could lead to stiffer lower limbs and reduce joint stability and balance [34]. The results of the aforementioned studies are consistent with the current research.

The ankle strategy is essential to maintaining balance in case of an abrupt disturbance in the body and the shifting of the gravity line as it returns the center of gravity to its original position. It is believed that the ankle strategy is effective in maintaining the static control of posture by adjusting the center of gravity [35]. In fact, the sudden giving way, shifting, and slipping of the knee in KOA patients is similar to a disturbance that abruptly takes the direction of the line of gravity off the base of the support and could be considered a hypothesis to upset the balance of these patients. Therefore, the weakness of the muscles acting on the ankle, the knees, and the back could explain the disorder in knee instabilities and the poor balance of these patients. In a similar study, Nevitt et al. (2016) investigated knee joint instability as a risk factor for falls in patients with KOA, reporting that with decreased KI, the number of falls decreased as well [36]. In the mentioned study, a questionnaire was used to assess balance, which was significantly different from the research methodology of our study. On the other hand, Sanchez-Ramirez et al. (2013) reported different results and observed no association between postural control and KI in osteoarthritis patients. This discrepancy could be attributed to the different tests used to assess balance in the mentioned study [16].

Whether as a fall experience or a feeling of KI, poor balance is associated with decreased daily performance, even in those who have not experienced a fall [30, 36]. Despite the major complaints of osteoarthritis patients of frequent knee giving way and instabilities, these issues have mostly been disregarded by specialists and physiotherapists, and the cause remains unidentified. In addition, it is unclear whether instability is the cause of imbalance or poor balance is the cause of instability. Knee instability is a multifactorial condition, which may occur due to increased capsular-ligament laxity, damage to the knee structures, decreased muscle strength, altered muscle activity patterns, and altered neuromuscular control, Therefore, insufficient joint stability is provided during daily activities and this leads to pain, swelling, and decreased mobility, and ultimately affects the patient's posture control and balance [3, 4, 37, 38]. In a systematic review in this regard, Wallace et al. (2019) reported that knee instability may occur due to the proprioception impairment or lack of posture control as the patient is unable to be clearly positioned and control the joint movement. However, this hypothesis has not been proven [2], the finding is consistent with the results of the present study. However, our findings indicated no significant differences between the KOA patients with and without KI in terms of TSK, which may be due to psychological impairment and the subjective assessment of the fear of movement. Therefore, further investigations should be more focused on the psychological impairments associated with KOA with and without KI.

Our findings indicated no significant differences between osteoarthritis patients with and without KI in terms of the right and left gait line length indices and foot contact time. No studies have compared the gait pattern between patients with osteoarthritis with and without KI. Nevertheless, the findings regarding the gait mechanics in KOA patients have shown a correlation between disease severity and gait mechanics although it is rather debatable, especially when adding the characteristic of joint instability to the problem. In this regard, Na and Buchanan reported that the exacerbation of KOA impairs the gait parameters as it increases the activation of some muscles compared to the patients with less severe KOA [39]. Depending on the level and severity of the disease, the signs and symptoms of KOA may affect the gait pattern of the patients (e.g., walking). Gait parameters in patients with knee OA during level walking have been characterized by slower walking velocity, lower cadence, shorter step length, longer stride time, and longer double-support time [40].

Elderly patients with KOA change their gait pattern to reduce the load on the joint while walking, which is accompanied by changes in the kinetic and kinematic gait parameters, such as speed, bending angle, and knee extension torque. These changes, in turn, may affect the status of accepted patterns compared to healthy individuals at the beginning of walking [41]. Gait initiation is characterized by a change from the static to the dynamic state, which poses challenges to the systems that are responsible for controlling the posture and is intended as an indicator of balance. It seems that the compensatory strategies of these patients are impaired in adapting to walking, thereby reducing their ability to walk properly.

According to the literature, KOA patients have a greater step width (28%), a smaller number of steps (11%), and a shorter step length (7%) compared to healthy individuals, which is mainly due to pain and stiffness [41, 42].

Another finding of the current research demonstrated positive and significant correlations between pain intensity, disease duration, and the rate of self-report instability in the women with KOA. Evidence attests to the effect of age on multiple sensory inputs, as well as the musculoskeletal system and the ability of the central nervous system to perform sensory-motor integration. With increased pain intensity and disease duration, the rate of KI increases in KOA patients. In a similar study, Fitzgerald et al. (2004) stated that repeated periods of KI caused excessive shear forces in the joint, which may accelerate disease progression. Therefore, the self-reported KI is associated with increased pain intensity, more falls, and changes in the gait pattern [27]. In another study, Lamba et al. (2018) also reported that if therapeutic interventions are not performed in a timely manner, the progression of KOA over time and disease duration cause the symptoms of KI to intensify [43]. Our findings also showed that the increased duration of the disease and disease deterioration increased the risk of falling and KI.

Also, when the knee flexion angle was compared between groups in both legs, we found that there was a significant difference between the two groups. This can have two causes (1). Due to the different pain intensity that was higher in the KI group, so this was due to the inability to bend the knee. (2) It was due to the knee contracture flexion, which should be evaluated in future studies.

Our study had some limitations, such as the lack of the kinematic study of the gait in the study groups and their comparison in terms of kinematic gait patterns, the impossibility of evaluating the electrical activity of the lower-extremity muscles during gait and the comparison of the patients with and without KI, the impossibility of assessing muscle strength and the correlations between the KI with the quadriceps and hamstring strength, and the absence of male patients in the sample population due to the religious restrictions of the Islamic Republic of Iran.

## Conclusion

Mean comparison showed a significant difference between the subjects with and without KI in static balance only in anterior–posterior direction with open eyes and closed eyes. In the dynamic balance test, the subjects in both groups had significant differences in terms of all the indicators of anterior–posterior stability, medial–lateral stability, and overall stability with closed eyes. However, no significant difference was observed with open eyes. Additionally, no significant differences were observed between the two groups in terms of the duration of foot contact and the length of the step. Multiple regression also indicated significant positive correlations between pain intensity and disease duration with the degree of KI.

The findings of this study could provide useful information on the postural sways and balance status of KOA patients with and without KI since balance is considered a significant risk factor for falling in these patients. Unlike previous studies, which investigated the balance of patients using the Berg balance scale, we measured balance using the most reliable device for this purpose (Biodex balance system), which provides beneficial data to experts and researchers. Further investigations in this regard should continue this important work to identify the root causes of KI and adopt proper strategies to control this disorder. Suggested measures include strengthening the knee muscles, improving knee proprioception, reducing knee contracture flexion, and reducing knee joint stiffness.

## Data Availability

The datasets collected during the current study are available from the corresponding author upon reasonable request.

## Abbreviations

KI: Knee instability
KOA: Knee osteoarthritis
BBS: Biodex balance system
VAS: Visual analog scale
AP: Anterior–posterior
ML: Medial–lateral
ICC: Inter correlation coefficient
APSI: Anteriorly-posteriorly stability index
MLSI: Medially-laterally stability index
OSI: Overall stability index
TSK: Tampa scale of kinesiofobia
